# Expression Analysis of Sugarcane Aquaporin Genes under Water Deficit

**DOI:** 10.1155/2013/763945

**Published:** 2013-12-29

**Authors:** Manassés Daniel da Silva, Roberta Lane de Oliveira Silva, José Ribamar Costa Ferreira Neto, Ana Carolina Ribeiro Guimarães, Daniela Truffi Veiga, Sabrina Moutinho Chabregas, William Lee Burnquist, Günter Kahl, Ana Maria Benko-Iseppon, Ederson Akio Kido

**Affiliations:** ^1^Department of Genetics, Federal University of Pernambuco (UFPE), 50670-901 Recife, PE, Brazil; ^2^Biotechnology Division, Sugarcane Technology Center (CTC), 13400-970 Piracicaba, SP, Brazil; ^3^Institute of Molecular Biosciences, Frankfurt University, 60438 Frankfurt am Main, Germany

## Abstract

The present work is a pioneer study specifically addressing the aquaporin transcripts in sugarcane transcriptomes. Representatives of the four aquaporin subfamilies (PIP, TIP, SIP, and NIP), already described for higher plants, were identified. Forty-two distinct aquaporin isoforms were expressed in four HT-SuperSAGE libraries from sugarcane roots of drought-tolerant and -sensitive genotypes, respectively. At least 10 different potential aquaporin isoform targets and their respective unitags were considered to be promising for future studies and especially for the development of molecular markers for plant breeding. From those 10 isoforms, four (*So*PIP2-4, *So*PIP2-6, *Os*PIP2-4, and *S*sPIP1-1) showed distinct responses towards drought, with divergent expressions between the bulks from tolerant and sensitive genotypes, when they were compared under normal and stress conditions. Two targets (*S*sPIP1-1 and *So*PIP1-3/PIP1-4) were selected for validation via RT-qPCR and their expression patterns as detected by HT-SuperSAGE were confirmed. The employed validation strategy revealed that different genotypes share the same tolerant or sensitive phenotype, respectively, but may use different routes for stress acclimation, indicating the aquaporin transcription in sugarcane to be potentially genotype-specific.

## 1. Introduction

Sugarcane (*Saccharum* spp.) is a valuable crop once it accumulates high levels of sucrose in the stems [1, 2]. In 2011, the twenty largest sugarcane producers generated about 1.7 billion tons of sucrose worldwide, valued about 52.5 billion dollars [3]. However, abiotic stresses can reduce the potential yield of these cultivated plants by 70%, with drought being the most dangerous one [4]. Water deficit, and its influence onto a variable number of morphological and functional characters in plants, eventually becomes one of the main obstacles to sustainable agricultural production worldwide [5].

The reduction of the water content in a plant cell provokes a complex network of molecular responses, involving stress perception, signal transmission in a transduction cascade and physiological, cellular, and morphological changes [6], including stomatal closure, suppression of cell growth and photosynthesis, and activation of cellular respiration. Plants under drought still respond to it and adapt by accumulating specific osmolytes and proteins for stress tolerance [7].

Genes expressed during drought can be classified into two functional groups. The first group encodes proteins that increase plant tolerance to stress, such as water channels proteins (aquaporins), proteases, and detoxification enzymes, all having a protective function. To this group belong enzymes catalyzing the biosynthesis of osmolytes, like derivatives of amino acids, sugars and various LEA (Late-Embryogenesis-Abundant) proteins. The second group of genes encodes various proteins, such as transcription factors, kinases, phosphatases, and enzymes involved in regulatory pathways, as phospholipid metabolism and ABA biosynthesis [7]. The aquaporins or MIPs (Major Intrinsic Proteins) are proteins assembling into water channels of cell membrane and facilitate osmosis for rapid bidirectional transport of water [8]. Besides, these proteins are also involved in many plant metabolic processes, including acquisition of nutrients, cell growth, carbon fixation, cell signaling, and various stress responses [9, 10]. The aquaporins also allow permeation of small molecules such as glycerol [11], urea [12] CO2 [13], ammonia [14], boric acid [15], H2O2 [16], and even arsenic [17]. According to the phylogenetic analysis of Johanson and Gustavsson [18], plant aquaporins are classified into four main subfamilies, widely distributed among higher plants: PIPs (plasma membrane intrinsic proteins), TIPs (tonoplast intrinsic proteins), SIPs (small basic intrinsic proteins), and NIPs (26 kDa intrinsic proteins). The aquaporins are presently and extensively studied, since their importance spans from animal [19] and human physiology [20] to osmo-adaptation of microorganisms [21] and vegetables [22, 23]. The transcripts encoding sugarcane aquaporins have only marginally been described, despite their significant physiological influence and participation in several processes during plant growth and acclimation against biotic and abiotic stresses [24, 25].

The present study is a first attempt to derive expression markers (functional molecular markers) from HT-SuperSAGE transcriptional profiles in contrasting sugarcane genotypes, in particular addressing specific sugarcane aquaporins, with the aim of better understanding the molecular processes occurring during drought response of the plant. HT-SuperSAGE, among all the genome-wide transcriptome profiling techniques was chosen for its efficiency to generate highly reliable transcription profiles. The increase in the size of the tag to 26 bp, the characteristic of SuperSAGE, drastically improves the annotation of the tag to the corresponding gene [28], allowing to establish genome-wide gene expression profiles of two or more species in one sample (e.g., host-parasite interactions [29, 30]). Besides, SuperSAGE combined high-throughput next-generation sequencing [31, 32], designated DeepSuperSAGE or HT-SuperSAGE, provides even more informations (three to four orders of magnitude) at relatively low cost compared to traditional Sanger sequencing.

## 2. Methodology

### 2.1. Unitags Annotation, GO Categorization of ESTs, and Aquaporin Isoforms Identification

Bioinformatics analyses covered the 8,787,315 tags (26 bp) described by Kido et al. [33] from four root HT-SuperSAGE libraries [SD24T (the bulk of the tolerant genotypes CTC6, CTC15, SP83-2847, SP83-5073, under stress (24 h of continuous dehydration), totalizing 2,542,552 tags); SDTC (the tolerant bulk with daily irrigation, comprising 1,909,543 tags); SD24S (the sensitive bulk of stressed genotypes CTC9, CTC13, SP90-3414 and SP90-1638, with 2,170,998 tags) and SDSC (the bulk of sensitive genotypes without stress, covering 2,164,222 tags)]. After singlet exclusion (tags sequenced only once per library), the unique tags (unitags) were classified as up- (UR) or down-regulated (DR), based on the Audic and Claverie test (*P* < 0.05; [34]), using the DiscoverySpace 4.0 software [35]. The unitag frequencies normalized to a million per library allowed the evaluation of the unitag expression modulation by fold change values (FC) comparing two frequencies. The unitags were aligned by BLASTn with expressed sequence tags (ESTs) from nine public databases, comprising sugarcane ESTs from NCBI (http://www.ncbi.nlm.nih.gov/nucest), and grass ESTs (Poaceae family) from Gene Index (http://compbio.dfci.harvard.edu/tgi/plant.html), including *Saccharum officinarum* (SoGI 3.0), *Sorghum bicolor* (SbGI 9.0), *Zea mays* (ZmGI 19.0), *Oryza sativa* (OsGI 18.0), *Panicum virgatum* (PaviGI 1.0), *Triticum aestivum* (TaGI 12.0), *Hordeum vulgare* (HvGI 11.0), and *Festuca arundinacea* (FaGI 3.0). Only BLASTn alignments (*e value* < 0.0001) with scores 42 to 52 (100% identity), plus/plus orientation and a preserved 5′CATG were accepted, and the best tag - hit was selected prioritizing sugarcane sequences or sequences from closely related species with adequate annotation. ESTs anchoring unitags were then categorized via Gene Ontology (GO; http://www.geneontology.org/GO.doc.shtml), using the Blast2GO tool [36].

Potential ESTs from the MIP gene superfamily were identified using the keywords “aquaporin,” “major intrinsic protein,” “PIP,” “TIP,” “PIN,” “SIP,” “plasma membrane intrinsic protein” “tonoplast intrinsic protein,” “nodulin-26-like intrinsic protein” and “small basic intrinsic protein” in the EST annotations, or “water transporter” in the GO terms. These ESTs were classified into the plant aquaporin subfamilies (TIP, NIP, SIP, PIP) and analyzed with the NCBI Conserved Domain Search tool (http://www.ncbi.nlm.nih.gov/Structure/cdd/wrpsb.cgi) in an effort to confirm their conserved domains. Also ESTs were BLASTx aligned with proteins sequences from the UniProtKB/Swiss-Prot database (http://www.uniprot.org/help/uniprotkb), trying to confirm the isoform identity by using curated sequences (*e*-value cutoff e^−10^).

### 2.2. Comparative and Phylogenetic Analysis of the Putative Aquaporin Isoforms Based on Unitag Expressions

The predicted peptides from ESTs related to the tags after translation with the ORF finder tool (http://www.ncbi.nlm.nih.gov/projects/gorf/) and BLASTp analysis were subsequently aligned via Clustal W [37]. A dendrogram of the aligned sequences was generated using the MEGA v.5.2.1 software [27], according to the following parameters: Neighbor Joining tree method, pairwise deletion option, and 1000 bootstrap replicates. For a better assignment of the isoforms into the aquaporin subfamilies, 15 *A. thaliana* aquaporin protein sequences were included in addition to the predicted proteins. Also, two sequences served as outgroups, one from humans and one from *Yersinia pseudotuberculosis*. In addition to the phylogenic tree, a heat map was established based on fold changes of the unitags responding to the applied stress.

### 2.3. cDNA Synthesis, Primer Design, and RT-qPCR Analysis

The RNA of each genotype sample constituting the bulks (tolerant stressed, tolerant control, sensitive stressed, and sensitive control) was isolated from sugarcane roots using the RNAeasy Plus Micro Kit (Qiagen) and DNase treatment. The cDNA synthesis reaction was performed with the SuperScriptTM First Strand kit Synthesis System for RT-PCR (Invitrogen) according to the manufacturer's protocol, using 1 g of RNA quantified by the Qubit fluorometer (Invitrogen). Primer pairs were designed from ESTs anchoring unitags, tolerating a maximum of one mismatch, and using the default parameters of the *Primer3* software [38] with some minor modifications [amplicon size: 70 to 200 bp; primer Tm: 40 to 60°C; GC content: 45 to 55%]. These ESTs anchored unitags differentially regulated in the tolerant and sensitive genotype bulks. Prior to the validation of unitags by RT-qPCR, standard curves using a dilution series of the cDNA pool were made to calculate the gene-specific PCR efficiency and regression coefficient (*R* ^2^) for each gene (Table 6). The RT-qPCR amplifications were performed on the LineGene 9660 model (Bioer), using SYBR Green detection. Each reaction mixture comprised 1 *μ*L of template cDNA (diluted 5-fold), 5 *μ*L of *HotStart-IT SYBR Green qPCR Master Mix 2x* (USB), 0.05 *μ*L of ROX, 1.95 *μ*L of water, and 1 *μ*L primer (500 nM each) to a final volume of 10 *μ*L. The reactions were denatured at 95°C for 2 min, followed by 40 cycles of 95°C for 15 s, 58°C for 30 s, and 72°C for 15 s in 96-well reaction plates, with the detection of the fluorescence signal at the end of each extension step. The melting curves were analyzed at 65–95°C for 20 min after 40 cycles. Three biological replicates and three technical replicates were used for RT-qPCR analysis. The relative quantification data were analyzed with the REST^©^ v.2.0.13 software [26].

## 3. Results and Discussion

### 3.1. Aquaporin Data-Mining and Categorization of EST Anchoring Unitags

The universe of 8,787,315 tags (26 bp) generated from the four HT-SuperSAGE libraries presented 205,975 unique tags (unitags) [33], from which 289 anchored in 484 putative aquaporin ESTs, distributed in nine different databases (details in Table 1), totaling 1,579 BLASTn results with scores of 42 to 52 (100% identity). This set of 484 ESTs anchoring unitags (Table 1) represented the basis for the analysis of transcript profiles based on the respective unitags.

**Figure 1:**
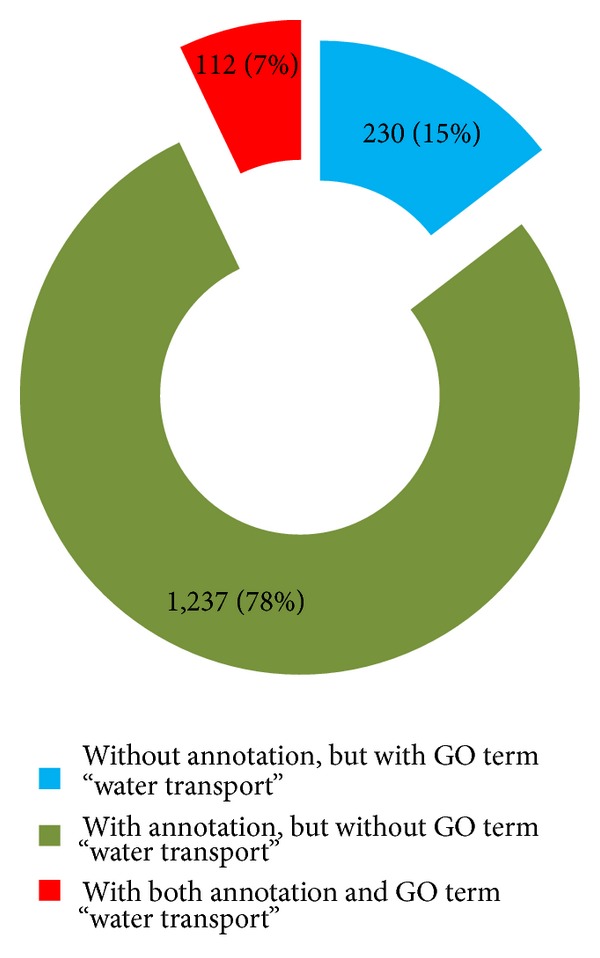
Percentage of HT-SuperSAGE unitags from sugarcane plants (24 h of continuous dehydration or regular daily irrigation) identified as potential aquaporins after keyword searches in the EST annotation (“*aquaporin*,” “*tonoplast intrinsic protein*,” “PIP,” “TIP,” “NIP,” “SIP”) or in the GO terms (“*water transport*”). Total of unitags: 1,579.

The keyword (“aquaporin,” “major intrinsic protein,” “tonoplast intrinsic protein,” “plasma membrane intrinsic protein,” “small basic intrinsic protein,” “nodulin-26-like intrinsic protein,” “PIP,” “TIP,” “NIP,” “SIP”) searches in the EST annotation identified 1,347 ESTs, while the “water transport” GO expression identified 342 ESTs (Figure 1). The searches in the GO terms increased the aquaporin identifications by almost 15%, representing 230 alignments of the total (1,579; Figure 1).

The unitag annotation efficiency relied on the used EST database. As mentioned by Kido et al. [39], Gene Index is a good source for unitag annotation, as it displays adequate gene or protein function descriptions. In the present case, the SoGI (*Saccharum officinarum* L.) dataset representing 282,683 ESTs that resulted in 121,342 unique sequences [42,377 TC (Tentative Consensus clusters) plus 78,965 singletons) after clustering. This species took part in the sugarcane breeding programs [40, 41] performed around the world.

Almost all unitags related to the aquaporin annotations (260 of 289; Table 1) anchored in SoGI sequences, which exceeded those obtained with the partial dbEST dataset (http://www.ncbi.nlm.nih.gov/dbEST/dbEST_summary.html) by almost six times. This dbEST is composed of 277,266 ESTs, mostly from Brazilian sugarcane hybrids (SUCEST-FAPESP project) [42] (Table 1). This may mainly be due to the sizes of the SoGI sequences, as most alignments occurred in the CTs. Nevertheless, the partial dbEST dataset was the second best source for mapping unitags, but its real annotation power was affected by the nonadequate descriptions of the cDNAs (many “unknown” hits). As Sorghum bicolor is the most closely related diploid of *S. officinarum* [2], this species could contribute to the identification of aquaporin isoforms. However, after redundancy exclusion, only 19 unitags anchored in seven unique ESTs based on the best hits (Table 1). This poor performance may be explained by the low number of ESTs available in the SbGI dataset (46,043), being the second smallest databank used (Table 1), in contrast with the high number of available sugarcane ESTs, reinforced by the high homology between sorghum and sugarcane.

The unitags proved to be highly specific for aquaporins. A total of 263 unitags (91% of 289) were associated with aquaporin isoforms (189 unitags anchored in just a single EST from a unique database); 19 unitags (7%) were not isoform-specific but comprised the same subclass (PIP1 or PIP2) and only seven (2%) were not specific to any subclass.

### 3.2. Comparative Analysis of the Putative Aquaporin Isoforms Identified by the Unitags

Regarding the total of 484 annotated aquaporin-ESTs anchoring unitags (Table 1), 470 of them (97.10%) aligned with aquaporin-proteins isoforms with an *e* value < 10^−20^ (BLASTx), from the UniProtKB/Swiss-Prot database, a high-quality annotated databank. This fact confirmed the isoforms identities (data not shown). All of them represented 42 distinct isoforms belonging to the four subfamilies (PIP, TIP, SIP, and NIP) based on the ESTs annotations. The 42 isoforms and their respective number of unitags [considered UR, DR or n.s. (*P* < 0.05)] in the two main HT-SuperSAGE libraries contrasts are shown in Table 2. According to this table, one unitag or more could be associated with a specific isoform. In some cases, two or more ESTs from one database present the same isoform annotation. The Gene Index databases used throughout this work minimized this situation due to the assembled TC (Tentative Consensus) clusters. Besides, unitags aligning more than one locus in the same EST could be resulted by partial *Nla*III digestions. In an attempt to avoid this situation, it was performed double digestions. Additionally, this event could be resulted by sister-tags anchoring one specific EST and isoform. In this case, tags showing a single base substitution (sister-tags) were considered as two different unitags. On the other hand, alternative transcripts could anchor varied unitags. Also, specific isoforms could be mapped in several loci (in the same or in different chromosomes). In addition, *Saccharum* hybrids show complex genomes, as a result of polyploidy and aneuploidy events [40, 41]. Therefore, this diversity of unitags (UR and DR) associated to aquaporin isoforms could allow identify biotechnologically interesting candidates.

**Figure 2:**
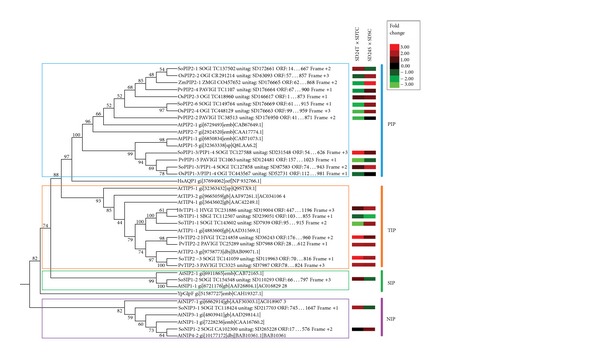
Neighbor Joining dendrogram (MEGA v.5.2.1 software [27]) and integrated heat map (bootstrap values of 1,000 replications), showing the phenetic grouping of 24 potential aquaporin amino acid sequences identified by HT-SuperSAGE unitags from sugarcane accessions after 24 h of continuous dehydration (with their respective EST and unitag identifiers), and aquaporins sequences of *Arabidopsis thaliana*, human and *Yersinia pseudotuberculosis* (all these labeled by an asterisk). Nomenclature: Isoforms are preceded by the abbreviated species name (*At-Arabidopsis thaliana; Hs-Homo sapiens; Hv-Hordeum vulgare; Os-Oryza sativa; Pv-Panicum virgatum; Sb-Sorghum bicolor; So-Saccharum officinarum; Yp-Yersinia pseudotuberculosis; Zm-Zea mays*).

From the 71 unique ESTs involved in perfect unitag-EST BlastN alignments (score 52; Table 1), 24 putative aquaporins showed ORFs with over 180 amino acids in size, and these sequences, together with MIP protein sequences from *Arabidopsis thaliana*, *Homo sapiens*, and *Yersinia pseudotuberculosis* were compared in a phenetic analysis. The resulting tree confirmed that putative aquaporins clearly divide into four major clusters, representing the PIP, TIP, SIP, and NIP subfamilies (Figure 2). This tree was consistent with a previous analysis of aquaporin phylogeny in higher plants [10, 18, 43, 44]. As expected, the human HsAPQ1 isoform grouped with the PIP subfamily, since the human APQ1 subfamily was recently recognized to be phylogenetically more similar to the PIP subfamily than to other plant subfamilies [45]. Also, YpGIpF grouped with the NIP aquaporin subfamily. The YpGIpF isoform belongs to a MIP family related to the bacterial GlpF protein glycerol uptake facilitator, classically associated with aquaglyceroporins from NIP and APQ3 subfamilies [45]. Therefore, this tree, which is supported by the scientific literature, presents the 24 aquaporin isoforms identified by HT-SuperSAGE unitags expressed after 24 h of continuous dehydration stress. Moreover, considering only the nine isoforms identified from *S. officinarum* ESTs, this smaller set was also distributed across the four aquaporin subfamilies described for higher plants.

Additionally, the heat maps (Figure 2) revealed by the expression modulation of the unitags (FCs) in the tolerant or sensitive bulks (both with their respective unstressed controls) show that some PIP isoforms are divergently regulated in the bulks of genotypes. Thus, from the 12 PIP transcripts, eight were repressed in the tolerant genotypes under stress. At the same time, eight of those transcripts were induced in the sensitive bulk. Furthermore, the majority of the PIP transcripts showed divergent modulations (contrasting results) when the response of both bulks of stressed genotypes is compared. Taken together, different genotypes may have developed different survival strategies.

On the other hand, the TIP transcripts similarly responded to the stress (comparing the modulation between both bulks of genotypes, Figure 2). Of the seven TIP subfamily isoforms studied (Figure 2), five were induced in both bulks of genotypes responding to stress, suggesting the participation of these isoforms in water transport. Finally, the only SIP subfamily representative studied here showed distinct regulation between the analyzed bulks, whereas the two NIP subfamily representatives distinctly responded: NIP3-1 was induced in the tolerant bulk and suppressed in its sensitive counterpart, while NIP3-2 was not modulated in the tolerant bulk, but was induced in the sensitive bulk (Figure 2). For these subfamilies, a larger amount of data is required for further analysis.

### 3.3. Transcriptional Profile of Putative Aquaporins Based on Unitags

The 30 most expressed unitags, based on their normalized frequencies (tpm) in the HT-SuperSAGE libraries, associated to the aquaporin subfamilies PIP (15), TIP (10), SIP (3) and NIP (2) are displayed in Table 3. According to Kjellbom et al. [46] many aquaporin genes are constitutively expressed, with a large number of transcripts (as presented in Table 3), while others are temporally and spatially regulated during plant development or stress responses, as is, for example, the case with unitag SD173276 (Table 3).

**Figure 3:**
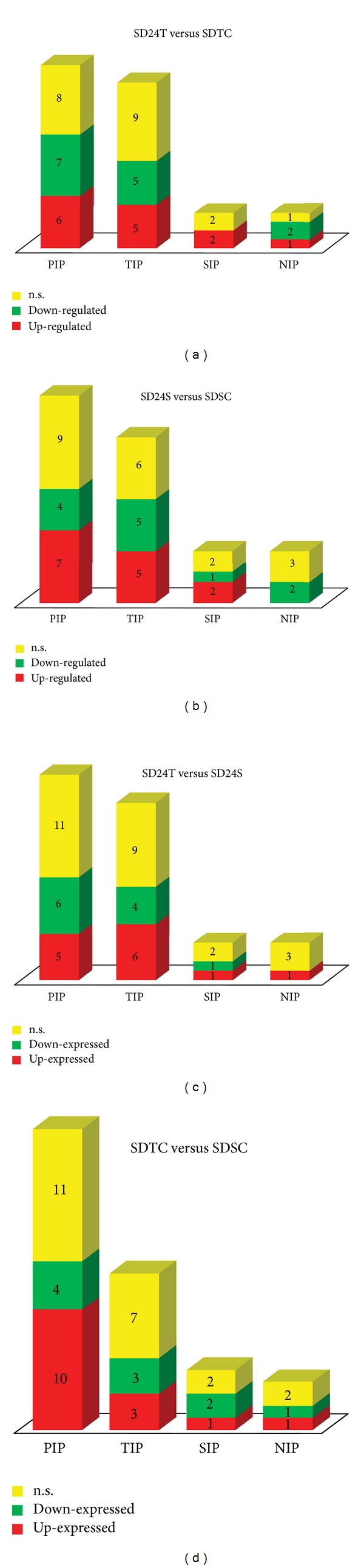
Representation of aquaporin subfamilies expressed in sugarcane after 24 h of continuous dehydration ((a), (b), and (c)) and during normal irrigation (d), involving bulks of genotypes tolerant and sensitive to stress. HT-SuperSAGE libraries: SD24T (bulk of tolerant genotypes under stress; SD24S (bulk of sensitive genotypes under stress); SDTC (control bulk of tolerant genotypes); SDSC (control bulk of sensitive genotypes).

After necessary redundancy exclusion, we identified 42 potential aquaporin isoforms. The contribution of each aquaporin subfamily is presented in Figure 3. In each comparison (SD24T vs SDTC: 26; SD24S vs SDSC: 28; SD24T vs SD24S: 28; SDTC vs SDSC: 28), 26–28 isoforms were identified as being expressed in sugarcane roots after onset of stress (24 h of continuous dehydration) or under normal daily irrigation conditions. This number of isoforms is close to that of other higher plants (31 aquaporin isoforms in maize [43, 47], 35 in *A. thaliana* [48], 39 in rice [47]), and more than twice the amount predicted for vertebrates (11 to 13 isoforms) [49, 50]. The number of aquaporin isoforms in sugarcane may be even higher, since some isoforms respond only in specific tissues [51] or after - salinity [52], freezing [53], mycorrhization [54], light [55, 56], and cell growth stresses [10]. Therefore, the real number of aquaposin isoforms can only be estimated approximatively by whole genome sequencing. Since sugarcane has one of the most complex genomes of the plant kingdom, with a diploid number of chromosomes ranging from 100 to 130 as a result of aneuploidy and polyploidy events [41], this approach would require significant efforts and investments.

The most transcribed 19 aquaporin unitags belonged to the PIP and TIP subfamilies (Table 3), which matches a report by Alexandersson et al. [51], who analyzed the transcriptional profile of 35 *Arabidopsis* aquaporins in three different tissues (roots, leaves, and flowers) during water deficit stress (watering suppression). These authors concluded that in all the studied tissues, the PIP, and TIP aquaporins showed higher expression levels, whereas NIPs aquaporins exhibited particularly low transcriptional levels under stress. Zhu et al. [52] also confirmed a lower amount of NIP and SIP in corn under controlled conditions (continuously aerated hydroponic medium, and parameters described by Gibeaut et al. [57]), as compared to the PIP and TIP, which could be related to the aquaporin transport specificity [58]. NIPs are related to the transport of small solutes [31], whereas the physiological functions of SIPs, in addition to water transport [59], still remain unclear. Otherwise, PIPs form primary channels mediating efficient water uptake and thereby control plasma membrane potentials of permeability, while TIPs, in addition to their high water transport capacity in tonoplasts [60], also transport CO2 [13] and urea [12].

In the present work, SIP and NIP subfamilies were less responsive to the applied stress. We noticed, that NIPs were not up-regulated in the sensitive bulks t SD24S versus SDSC (Figure 3(b)), as well as among the down-regulated unitags in the tolerant bulks SD24T versus SD24S (Figure 3(c)). On the other hand, the SIP subfamily also harbored no isoform among the down-regulated unitags in the contrast SD24T versus SDTC (Figure 3(a)). Alexandersson et al. [51] also confirmed that some SIP isoforms presented little expression variation in *Arabidopsis* plants under drought stress (watering suppression extended until 12 days). Therefore, AtSIP1-1 was considered as constitutively expressed. This low responsiveness to water deficit can be explained by the unique location of these aquaporins in the endoplasmic reticulum [59], an organelle with tortuous structure and high surface-to-volume ratio with high demand for osmotic balance volume and, therefore, may not require the water transport mediated by aquaporins [59]. Thus, further studies are necessary to define SIP functions more clearly.

The level of aquaporin transcripts varied less than 10 times based on the unitags in the contrasts, except for the SD173276 unitag (a potential SoTIP2-2), which was almost two thousand times higher in the tolerant SDTC versus SDSC contrast, and almost 500 times in the sensitive contrast SD24S versus SDSC (data not shown).

Alexandersson et al. [51] also observed that most aquaporins do not alter their expression under water deficit stress, and no *Arabidopsis* aquaporin isoforms varied their expression more than twice until the seventh day of stress treatment.

The contrast analysis of the tolerant bulks defined four possible targets: one exclusively up-regulated PIP1-1 isoform (SD264077 unitag, FC 3.58), and three exclusively down-regulated PIP2-2s (SD176950 unitag, FC -2.34), PIP2-1 (SD176664 unitag, FC -1.73) and NIP1-1 (SD202395 unitag, FC -1.36).

In rice, the PIP1-1 isoform promoted salt stress tolerance [23], and it was involved in the rehydration after cooling stress in tolerant genotypes [61]. PIP1-1 overexpression conferred tolerance to water deficit in rice and to salt stress in transgenic *Arabidopsis* [62]. This isoform also responded to drought and daytime in grapevine [56]. The up-regulated SD264077 unitag, as a possible PIP1-1 isoform, was validated in the present work by RT-qPCR analysis, as detailed in the next chapter, and represents a potential target for further studies, including the development of molecular markers for marker-assisted selection in breeding (real-time PCR-assisted selection) [63] or cis-genesis (insertion of genes in different accessions of the same species [64]), already successfully applied by Joshi et al. [65]. These authors inserted resistance genes to apple scab under the control of the RubisCO promoter in varieties known to be susceptible to the pathogen.

Isoform PIP2-2 is down-regulated over four times in *Arabidopsis* under 12 days of drought [51] and in barley under salt stress [66]. As expected, it was also observed in the tolerant bulk analysis of the present study, showing FC -2.34. The subsequent PIP2-2 monitoring revealed that aquaporin expression increased sensitivity to salt stress in transgenic rice [67]. This point is relevant, since crosstalks involving shared pathways in response to drought and salinity stress are regular [68, 69]. Thus, this isoform, after appropriate RT-qPCR validation, could be useful as stress-indicator in breeding programs.

On the other hand, the potential usefulness of unitags related to PIP2-1, PIP2-5, PIP2-6, TIP1-1, TIP2-2, SIP1-1, and SIP1-2 in breeding programs still need to be confirmed. In relation to the PIP2-5 isoform (up- and down-regulated simultaneously in the present study, depending on unitags), Jang et al. [70] observed that overexpressing this aquaporin reduced drought tolerance of transgenic *Arabidopsis* and tobacco. The same group proposed that PIP2-5 expression influenced the transcription levels of other PIPs and H+-ATPases (enzymes that regulate the cytoplasmic pH in which levels of H+ interfere with the control of the opening and closing of the aquaporins channels known as the aquaporin gating [71]). Lembke et al. [25] also observed this isoform to be down-regulated under water deficit (72 hours of watering suppression), despite the detected induction via oligonucleotide array hybridization.

Therefore, for the tolerant bulk of genotypes, this isoform is expected to restrain its expression under root dehydration (24 h).

Basically, up- or down-regulation and constitutive expression were all observed in the contrast analysis of tolerant bulks, (except that down-regulation was not observed in the SIP subfamily; Figure 3(a)).

The sensitive bulk of genotypes also presented all three expression levels for each aquaporin subfamily (with the exception of the NIP subfamily, in which up-regulation was not observed; Figure 3(b)). The analysis of the sensitive bulks allowed the identification of only up-regulated [PIP2-2 (SD176665 unitag, FC 4.83), PIP2-4 (SD176663 unitag, FC 1.66), PIP2-6 (SD176669 unitag, FC 1.70)] or only down-regulated [PIP1-1 (SD264077 unitag, FC -4.56), NIP1-1 (SD202395 unitag, FC -2.00)] aquaporin isoforms. These exclusively up- or down-regulated isoforms, respectively, may represent a panel of markers based on real-time PCR, and suggesting high stress sensitivity. In this way, at least two candidates are particularly appealing: (a) PIP1-1, that was up-regulated (SD264077 unitag, FC 3.58) in the tolerant bulks and entirely differently regulated (SD264077 unitag, FC -4.56) in the sensitive bulks; (b) PIP2-2 isoform, which was exclusively down-regulated in the tolerant bulks (SD176950 unitag, FC -2.34) and up-regulated in the sensitive bulks (SD176665 unitag, FC 4.83). Thus, both isoforms are strong candidates for further research aiming at molecular marker development and cis-genesis. Finally, further studies are needed to determine the true meaning of each stress-responsive isoform.

When comparing both genotype bulks under stress (SD24T versus SD24S), all three expression levels (up- or down-regulation and constitutive expression) were observed for each aquaporin isoform subfamily. Notably, a specific isoform in the SIP subfamily was down-regulated in the tolerant bulk, but not in the sensitive bulk (Figure 3(c)). Two more isoforms are worth mentioning: PIP2-4 (SD176664 unitag, FC 3.64), which was more transcribed in the stressed tolerant bulk than in the stressed sensitive one, and PIP2-1 (SD176669 unitag, FC -1.13), which is being less transcribed in the stressed tolerant bulk as compared to the stressed sensitive bulk.

The unitag related to PIP2-4 (SD176664) was down-regulated in the tolerant bulks after onset of the stress, and it had no relevant expression changes in the sensitive bulks. Nevertheless, it was more expressed in the tolerant bulk when compared with the sensitive bulk, upon stress or even under control conditions. Thus, the tolerant genotypes seemed to produce more PIP2-4 transcripts than the sensitive genotypes. In maize, this aquaporin isoform was up-regulated after only two hours of salt stress, in which time the recovery phase of the osmotic potential falls [52].

In turn, the PIP2-1-related unitag (SD176669) behaved differently under stress and its reaction depends on the genotype (it was down-regulated as compared to the tolerant bulks, and up-regulated as compared to the sensitive bulks). When considering the contrast between both control and stressed bulks of genotypes, this unitag was better expressed in the sensitive bulk than in the tolerant one. By taking into account that this aquaporin isoform increases insensitivity to salinity [67] in transgenic rice and the fact that salinity and drought share many response pathways [68, 69], this isoform deserves further investigation.

Analysis of controls of the two different genotype bulks s showed that the aquaporin isoforms are present in all subfamilies and expressed in the three studied levels during normal daily irrigation (Figure 3(d)). Four isoforms [PIP1-2 (SD92576 unitag); PIP2-1 (SD176664 unitag); PIP2-4 (SD176663 and SD176950 unitags); PIP2-6 (SD176669 unitag)] presented significantly higher abundance in the tolerant bulk, while only one (NIP1-1) was less expressed in relation to the sensitive bulk. In turn, five isoforms (PIP2-2, PIP2-3, PIP2-5, PIP2-6, and TIP4-2) were similarly transcribed in both bulks, while another five isoforms (PIP2-1, PIP2-4, TIP2-2, TIP2-3, and SIP1-2) presented all the three expression levels. Differences in the transcriptional profiles of both controls bulks reinforce the expression modulation of genes presenting in the genotypes composing the bulks.

The two main comparisons SD24T versus SDTC and SD24S versus SDSC revealed that from a total of 18 up-regulated unitags in the tolerant bulks, eight were down-regulated in sensitive bulks, while from 22 other unitags, down-regulated in the tolerant bulks, eight were up-regulated in the sensitive bulks (Table 4). The same isoforms showing different expression levels (Table 4) can be explained by the similarities between aquaporins sequences, in part a consequence of the high level of duplicated plant MIP genes, which is higher than that observed in vertebrates, possibly reflecting the environmental pressures plants are exposed to [45, 49], and also the aneuploidy and polyploidy events observed in the *Saccharum* complex [41].

### 3.4. Unitag Expression Validation by RT-qPCR

The use of RT-qPCR for the confirmation of aquaporin gene expression changes in grass (maize [72] and sugarcane [25]) has already been reported. In the present work we attempted to determine which genotype was responsible for the bulk of expression in tolerant or sensitive genotypes. To that end, each genotype composing that bulk was independently tested by RT-qPCR analysis. Thus, two unitags [SD264077 (PIP1-1) and SD231548 (PIP1-3/PIP1-4)] considered UR in the tolerant bulk as well as DR and n.s. in the sensitive were selected for expression validation using two reference genes (25S rRNA and GAPDH), both reported to be suitable for sugarcane (Table 5). The relative expression results of the tolerant and sensitive genotypes for the two target genes are shown in Table 6, together with their respective unitag results.

**Figure 4:**
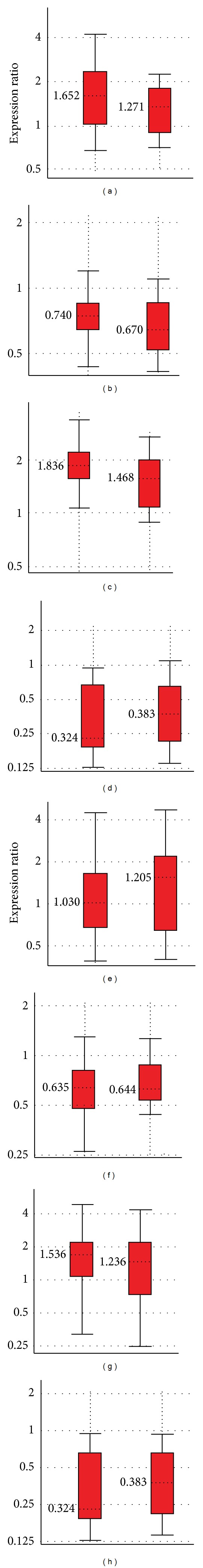
Relative quantification of SsPIP1 aquaporin-1 (unitag SD264077) and SoPIP1-3/PIP1-4 (unitag SD231548) with sugarcane cDNAs [tolerant genotypes: (a) CTC6, (b) CTC15, (c) SP83-2847, (d) SP83-5073; sensitive genotypes [(e) CTC9, (f) CTC13, (g) SP90-1638, (h) SP90-3414] under stress (24 h of continuous dehydration). The expression ratios of the same genotypes under control condition were normalized by reference genes GAPDH and 25S rRNA. ^#^Relative expression (REST^©^ v. 2.0.13 software) with the mean value (horizontally dotted line) and range of the observations (100%, horizontal bars); in red: confidence interval at 95%.

PIP1-1 (SD264077 unitag) was induced by stress in two of the tolerant genotypes (CTC6 and SP83-2847), in comparison to the respective controls (Table 6, Figure 4). Nevertheless, in the remaining tolerant genotypes (CTC15 and SP83-5073) both PIP1-1 genes were down-regulated under the same conditions (Table 6, Figure 4). Thus it can be concluded that CTC6 and SP83-2847 were responsible for the unitag up-regulation. The overexpression of rice PIP1-1 in root and leaf (within 24 h) enhanced the tolerance to drought (200 mM mannitol) and salt stress (100 mM NaCl) in transgenic *Arabidopsis* [62]. Also, PIP1-1 aquaporin isoforms in grapevine were highly expressed in roots (RT-qPCR) in response to water deficit (8 days of constant dehydration [56]).

PIP1-3/PIP1-4 (SD231548 unitag), were stress-induced in genotype SP83-2847 (Table 6, Figure 4), in agreement with the HT-SuperSAGE data. It should be noted that in phylogenetic analyses PIP1-3 and PIP1-4 are highly similar with barley PIP amino acid sequences, being grouped together as one isoform, while they are phylogenetically more distant from PIP1-1 (from barley and rice, [73]). *A. thaliana* PIP1-3 and PIP1-4 isoforms had their transcription level increased more than five times, covering the first 48 h of drought stress (250 mM mannitol), in leaves and roots, as well as in response to salt (150 mM NaCl) and cold stresses [48]. In turn, PIP1-3 overexpression in transgenic rice, combining aquaporin coding sequence with a constitutive corn promoter, showed enhanced stress tolerance to cold [74].

However, in relation to water transport by PIP1-3, which appears to be less permeable to water [74], this isoform could work best in conjunction with PIP2 subgroup members, in which *in silico* analysis showed them to be mostly DR. Considering the remaining components of the tolerant bulk, the genotype CTC6 did not show significant differences in transcript levels, while CTC15 and SP83-5073 presented down-regulated transcription at the onset of root dehydration (Table 6, Figure 4).

The strategy of opening bulks in the RT-qPCR validation reinforced the transcription modulation of sugarcane aquaporins and gave hints to genotype-specific expression. Thus, plants considered physiologically tolerant or sensitive to root dehydration (24 h) varied in the expression of aquaporin isoforms. The same was observed with up (*O. sativa* L. cv. Zhonghan 3) and lowland (cv. Xiushui) rice under water deficit [75]. The RT-qPCR results revealed genotype-specific differences for PIP1-2, PIP1-3, PIP2-1, and PIP2-5 isoforms in roots, and PIP1-2 and PIP1-3 in leaves. The above mentioned isoforms were up-regulated in upland rice, whereas they remained unchanged or DR in lowland rice [75].

Finally, the RT-qPCR protocol, in the present work applied for unitags validation, as well as the identified unitags for PIP1-1 and PIP 1-3/PIP1-4, define a set of functional molecular markers based on the expression profiles validated with appropriate genotypes. This expression marker set will assist breeders in marker-assisted selection of elite genotypes more tolerant to abiotic stresses.

## 4. Conclusions

The present work is a pioneer study specifically addressing the aquaporin transcripts in sugarcane transcriptomes established from HT-SuperSAGE transcription profiles from roots of tolerant and sensitive genotypes after 24 h of continuous dehydration. Almost all 26 bp unitags were annotated using a public sugarcane EST databases, especially S. by *S.*, allowing the identification of potential aquaporins. Categorizing the EST-anchored unitags by Gene Ontology (GO) enhanced the annotation efficiency by almost 15%. These procedures identified potential isoforms of the four aquaporin subfamilies (PIP, TIP, NIP, and SIP) already described for higher plants, together with their respective expression profiles in sugarcane under abiotic stress. Moreover, an efficient protocol for RT-qPCR was developed, enabling gene expression validation of SuperSAGE unitags related to PIP aquaporins (PIP1-1 and PIP1-3/PIP1-4) and involving reference genes encoding GAPDH and 25S rRNA, testing each genotype individually the employed, validation strategy revealed genotype-specificity of the response to the applied stress.

## Figures and Tables

**Table 1:** EST databases used for unitag annotations from sugarcane HT-SuperSAGE libraries (tolerant/sensitive genotypes after 24 h of continuous dehydration or regular daily irrigation) and BLASTn nonredundant results.

Species	Database	Annot.*/ESTs	PIP	TIP	NIP	SIP	Total BlastN Align.	Score 52	Align. (up to one mismatch)	Unitags	Unique ESTs^+^	Unitags with GO^1^
*Saccharum* spp.	dbEST	24/256,636	0	0	0	0	154	41	105	43	34	23
*Saccharum officinarum *	SoGI	265/121,342	157	85	21	11	683	158	425	260	127	50
*Sorghum bicolor *	SbGI	68/46,043	34	23	5	6	74	11	63	19	7	74
*Zea mays *	ZmGI	347/315,134	142	154	35	19	49	7	29	13	15	20
*Oryza sativa *	OsGI	283/201,220	121	95	33	7	119	26	72	20	86	3
*Panicum virgatum *	PaviGI	147/85,244	58	45	28	6	174	31	116	22	57	0
*Triticum aestivum *	TaGI	542/222,152	138	128	32	14	253	20	197	23	123	5
*Hordeum vulgare *	HvGI	110/83,101	32	33	12	9	34	2	30	16	21	7
*Festuca arundinacea *	FaGI	27/30,244	5	1	1	1	39	10	27	17	14	5

Total		1,913/1,361,144	687	564	167	73	1,579	306	1,064	289^#^	484	45^#^

*Aquaporin, tonoplast intrinsic protein and major intrinsic protein, membrane integral protein (PIP, TIP, NIP e SIP); ^+^number of nonredundant ESTs (putative aquaporins) anchoring unitags, ^1^“water transporter”; ^#^number without redundancy among the nine databanks.

**Table 2:** Putative sugarcane aquaporin isoforms (42) based on unitags of root dehydration (24 h) observed in the two main contrasts of HT-SuperSAGE libraries.

Aquaporin isoform	SD24T versus SDTC			SD24S versus SDSC		
	UR	DR	n.s.	UR	DR	n.s.
*S*sPIP1-1	1	—	2	—	1	1
*So*PIP1-2	1	2	3	2	1	—
*Pv*PIP1-2	—	—	1	—	—	1
*So*PIP1-3/PIP1-4	3	24	26	7	7	25
*Os*PIP1-3/PIP1-4	1	—	2	—	1	—
*So*PIP1-5	1	2	2	3	—	2
*Pv*PIP1-5	—	—	2	—	—	1
*So*PIP2-1	2	14	23	4	24	19
*Zm*PIP2-1	—	2	4	1	—	4
*Os*PIP2-1	1	—	2	—	1	2
*Pv*PIP2-1	—	1	9	1	—	6
*Ta*PIP2-1	—	2	4	1	—	2
*Os*PIP2-2	—	—	2	—	—	3
*Pv*PIP2-2	—	2	6	1	—	4
*Os*PIP2-3	—	—	1	—	—	1
*Zm*PIP2-3	—	—	1	—	—	1
*So*PIP2-4	3	4	6	2	3	6
*Os*PIP2-4	—	2	—	1	2	—
*Pv*PIP2-4	—	4	2	1	—	5
*Fa*PIP2-4	—	3	7	3	—	3
*So*PIP2-5	1	1	—	—	—	2
*Ta*PIP2-5	—	1	4	1	—	2
*So*PIP2-6	2	—	2	2	—	2
*Ta*PIP2-6	—	1	3	1	—	2
*S*sTIP1-1	4	9	14	6	5	18
*So*TIP1-1	3	7	10	6	7	11
*Sb*TIP1-1	1	—	1	—	1	1
*Zm*TIP1-1	—	—	1	—	—	—
*Hv*TIP1-1	1	—	—	—	1	—
*Ta*TIP1-1	—	1	4	—	1	4
*Ta*TIP1-2	—	1	—	—	—	1
*So*TIP2-2	2	9	9	1	8	11
*Hv*TIP2-2	1	—	1	—	—	1
*Pv*TIP2-2	—	—	2	—	—	2
*So*TIP2-3	5	5	10	3	3	5
*Pv*TIP2-3	—	—	3	—	—	3
*So*TIP4-2	—	—	1	—	—	1
*Pv*NIP1-1	—	1	—	—	1	1
*So*NIP1-2	—	—	1	—	—	1
*So*NIP3-1	1	2	12	—	1	10
*So*SIP1-1	1	—	2	1	—	1
*So*SIP1-2	3	5	2	2	1	10

Isoforms are preceded by the abbreviated species name (*Fa*: *Festuca arundinacea*; *Hv*: *Hordeum vulgare*; *Os*: *Oryza sativa*; *Pv*: *Panicum virgatum*; *Sb*: *Sorghum bicolor*; *So*: *Saccharum officinarum*; *Ss*: *Saccharum* spp.; *Ta*: *Triticum aestivum*; *Zm*: *Zea mays*). HT-SuperSAGE libraries: SD24T (bulk of tolerant genotypes under stress; SD24S (bulk of sensitive genotypes under stress); SDTC (control bulk of tolerant genotypes); SDSC (control bulk of sensitive genotypes). DR: down-regulated; UR: up-regulated. n.s.: not significant at *P* < 0.05.

**Table 3:** The 30 HT-SuperSAGE unitags most expressed and annotated as aquaporins from sugarcane libraries with contrasting genotypes under stress (24 h of continuous dehydration) or normal daily irrigation.

Unitag id	Aquaporin	Tags per million (tpm)			
		SD24T	SDTC	SD24S	SDSC
SD173282	*So*TIP2-2	1,096	3,784	1,643	1,816
SD231437	*So*PIP1-3/PIP1-4	819	1,551	1,140	990
SD87583	*So*PIP1-3/PIP1-4	964	956	1,186	520
SD119746	*So*TIP2-3	1,162	1,041	771	530
SD173276	*So*TIP2-2	564	1,879	496	0
SD182865	*So*TIP2-2	535	876	501	674
SD87593	*So*PIP1-3 /PIP1-4	579	601	750	377
SD80613	*So*TIP1-1	318	234	775	571
SD80612	*So*TIP1-1	437	393	423	453
SD250744	*So*PIP2-1	321	577	265	508
SD19004	*Hv*TIP1-1	422	395	411	210
SD176669	*So*PIP2-6	275	591	310	183
SD243880	*So*PIP2-1	176	326	334	496
SD28080	*So*PIP2-4	406	329	224	312
SD176663	*So*PIP2-6	135	340	227	136
SD241279	*So*PIP1-5	151	184	257	107
SD84960	*S*sTIP1-1	202	216	29	136
SD243849	SoPIP2-1	108	123	113	40
SD54852	*So*PIP2-1	107	143	22	52
SD96918	*So*SIP1-2	106	66	96	49
SD96922	*So*SIP1-2	89	43	114	49
SD202395	*Pv*NIP1-1	39	53	40	98
SD243867	*So*PIP2-1	37	42	79	43
SD87586	*So*PIP1-3/PIP1-4	31	54	28	55
SD250859	*So*PIP2-1	39	96	0	32
SD198883	*So*PIP2-5	39	27	30	37
SD21811	*So*SIP1-1	26	16	26	26
SD84958	*S*sTIP1-1	29	15	9	17
SD217703	*So*NIP3-1	29	15	13	13
SD36243	*Hv*TIP2-2	31	9	12	9

Isoforms are preceded by the abbreviated species name (*Hv*: *Hordeum vulgare*; *Pv: Panicum virgatum*; *So: Saccharum officinarum*; *S*s: *Saccharum* spp). SuperSAGE libraries: SD24T (bulk of tolerant genotypes under stress; SD24S (bulk of sensitive genotypes under stress); SDTC (control bulk of tolerant genotypes); SDSC (control bulk of sensitive genotypes).

**Table 4:** Aquaporin unitags with distinct expression rates in root HT-SuperSAGElibraries from contrasts of tolerant^T^ (SD24T versus SDTC) and sensitive^S^ (SD24S versus SDSC) sugarcane genotypes and after continuous dehydration (24 h).

Unitag	Anotation	FC^T^	Unitag regulation^T^	FC^S^	Unitag regulation^S^
SD264077	*S*sPIP1-1	3.58	UR	−4.56	DR
SD2444	*So*PIP1-3/PIP1-4	5.93	UR	−2.66	DR
SD231548	*So*PIP1-3/PIP1-4	3.18	UR	1.28	n.s.
SD243866	*So*PIP2-1	13.51	UR	−15.97	DR
SD243874	*So*PIP2-1	3.18	UR	−2.14	DR
SD28082	*So*PIP2-4	15.10	UR	−6.39	DR
SD28080	*So*PIP2-4	1.23	UR	−1.39	n.s.
SD198883	*So*PIP2-5	1.46	UR	−1.25	n.s.
SD36536	*So*TIP1-1	2.97	UR	−1.90	n.s.
SD80612	*So*TIP1-1	1.11	UR	−1.07	n.s.
SD84958	*S*sTIP1-1	2.02	UR	−1.82	DR
SD36243	*Hv*TIP2-2	3.53	UR	1.34	n.s.
SD182891	*So*TIP2-2	2.78	UR	−5.85	DR
SD119963	*So*TIP2-3	5.56	UR	1.93	n.s.
SD119859	*So*TIP2-3	3.18	UR	−2.66	DR
SD217703	*So*NIP3-1	1.93	UR	−1.01	n.s.
SD21811	*So*SIP1-1	1.65	UR	1.01	n.s.
SD96919	*So*SIP1-2	2.38	UR	−1.06	n.s.
SD233575	*So*PIP1-3/PIP1-4	−6.56	DR	4.21	UR
SD231438	*So*PIP1-3/PIP1-4	−5.16	DR	1.38	n.s.
SD231437	*So*PIP1-3/PIP1-4	−1.89	DR	1.15	UR
SD205705	*So*PIP1-3/PIP1-4	−1.87	DR	−1.14	n.s.
SD231440	*So*PIP1-3/PIP1-4	−2.79	DR	−2.14	n.s.
SD241279	SoPIP1-5	−1.22	DR	2.40	UR
SD91837	SoPIP2-4	−17.81	DR	1.32	n.s.
SD243847	*So*PIP2-1	−7.50	DR	−1.60	n.s.
SD243911	*So*PIP2-1	−5.62	DR	−1.33	n.s.
SD54851	*So*PIP2-1	−2.53	DR	1.87	n.s.
SD176663	*Os*PIP2-4	−2.51	DR	1.66	UR
SD176664	*So*PIP2-4	−1.73	DR	1.02	n.s.
SD84616	*So*PIP2-5	−4.42	DR	1.32	n.s.
SD176669	*So*PIP2-6	−2.15	DR	1.70	UR
SD19005	*Hv*TIP1-1	−1.87	DR	1.84	n.s.
SD19006	*Hv*TIP1-1	−5.16	DR	4.67	UR
SD7939	*Ta*TIP1-1	−2.95	DR	1.76	n.s.
SD80616	*S*sTIP1-1	−2.81	DR	2.34	UR
SD182871	*So*TIP2-2	−6.09	DR	−1.63	n.s.
SD173276	*So*TIP2-2	−3.33	DR	496.29	UR
SD119919	*So*TIP2-3	−6.56	DR	1.61	n.s.
SD194892	*So*NIP3-1	−2.34	DR	−1.60	n.s.

Isoforms are preceded by the abbreviated species name (*Hv*: *Hordeum vulgare*; *Os: Oryza sativa*; *So: Saccharum officinarum*;* S*s: *Saccharum* spp. and *Ta*: *Triticum aestivum*). HT-SuperSAGE libraries: SD24T (bulk of tolerant genotypes under stress; SD24S (bulk of sensitive genotypes under stress); SDTC (control bulk of tolerant genotypes); and SDSC (control bulk of sensitive genotypes). FC: ratio of the frequencies (normalized to 1,000,000) observed in the stressed library in relation to the control library. DR: down-regulated; UR: up-regulated; n.s.: not significant at *P* < 0.05.

**Table 5:** Primers sequences for RT-qPCR of *S*sPIP1-1, *So*PIP1-3/PIP1-4 (designed from sugarcane ESTs), 25S rRNA, and GAPDH (as reference genes).

Unitag	EST/cluster	Database*	Gene	Primers	Tm (°C)	Amplicon (bp)	Regression coefficient (*R* ^2^)	Amplification efficiency (%)
SD264077	gi∣35203438	dbEST	*S*sPIP1-1	5′-GTTCCTATCCTTGCCCCACT-3′ 3′-AGGCGTGATCCCTGTTGTAG-5′	84.6	134	0.995	95.55

SD231548	TC127588	SoGI	*So*PIP1-3/PIP1-4	5′-GACTCCCATGTTCCTATCCTTG-3′ 3′-CGTGATCCCTGTTGTAGATGAT-5′	84.3	142	0.992	93.47

—	gi∣33464288	dbEST	25S rRNA	5′-GCAGCCAAGCGTTCATAG-3′ 3′- CGGCACGGTCATCAGTAG-5′	82.9	172	0.999	99.82

—	TC531505	SoGI	GAPDH	5′-GGTTCACTTGAAGGGTGGTG-3′ 3′- TGAGGTGTACCTGTCCTCGTT-5′	81.8	100	0.984	100.89

Isoforms are preceded by the abbreviated species name (*So*: *Saccharum officinarum*;* S*s: *Saccharum* spp.).

*Databases: dbEST (NCBI; http://www.ncbi.nlm.nih.gov/), Gene Index (SoGI; http://compbio.dfci.harvard.edu/tgi/)].

**Table 6:** Relative expression rates of aquaporins PIP1-1 (SD264077 unitag) and PIP1-3/PIP1-4 (SD231548 unitag) in bulks of tolerant or sensitive genotypes, respectively, and RT-qPCR data (both in bulks and each genotype).

Technique and genotypes	Target gene	
	PIP1-1	PIP1-3/PIP1-4
	Modulation of gene expression^&^	
HT-SuperSAGE		
Tolerant bulk^1^	3.580*/UR	3.180*/UR
Sensitive bulk^1^	−4.560*/DR	1.280*/ns
RT-qPCR and Tolerant Genotypes		
CTC6	1.652^#^/UR	1.271^#^/ns
CTC15	0.740^#^/DR	0.670^#^/DR
SP83-2847	1.836^#^/UR	1.468^#^/UR
SP83-5073	0.324^#^/DR	0.383^#^/DR
RT-qPCR and sensitive Genotypes		
CTC9	1.030^#^/ns	1.205^#^/ns
CTC13	0.635^#^/DR	0.644^#^/DR
SP90-1638	1.536^#^/ns	1.236^#^/ns
SP90-3414	0.324^#^/DR	0.383^#^/DR

^1^Bulk of the four tolerant or sensitive genotypes; ^&^ *P* < 0.05;*fold change [FC: ratio of the frequencies (normalized to 1,000,000) observed in the stressed library in relation to the control library]; ^#^relative expression level using the REST software (v. 2.0.13) [26]; DR: down-regulated; UR: up-regulated; ns: not significant at *P* < 0.05.
